# Probiotic Bactolac alleviates depression-like behaviors by modulating BDNF, NLRP3 and MC4R levels, reducing neuroinflammation and promoting neural repair in rat model

**DOI:** 10.1007/s00424-025-03084-6

**Published:** 2025-04-26

**Authors:** Musab Işık, Fadime Köse, Özcan Budak, Cansu Özbayer, Rumeysa Keleş Kaya, Sevda Aydın, Aleyna Ceren Küçük, Mehmet Arif Demirci, Songül Doğanay, Cahit Bağcı

**Affiliations:** 1 Department of Physiology, İstanbul Aydın University Medical Faculty, Istanbul, Turkey; 2 Department of Physiology, Sakarya University Medical Faculty, Sakarya, Turkey; 3 Department of Hıstology-Embryology, Sakarya University Medical Faculty, Sakarya, Turkey; 4 Department of Medical Biology, Medical Faculty, Kütahya Health Sciences University, Kutahya, Turkey; 5 Department of Medical Pharmacology, University of Health Sciences Hamidiye International School of Medicine, Istanbul, Turkey; 6 Department of Health Systems Management, Muş Alparslan University, Faculty of Health Sciences, Muş, Turkey

**Keywords:** Chronic stress, Depression, Depressive-like behaviors, Neurodegeneration, Probiotic

## Abstract

Depression, a prevalent psychiatric disorder, exerts severe and debilitating impacts on an individual's mental and physical well-being, and it is considered a chronic mental illness. Chronic stress plays an important role in the pathophysiology of depression. *Lactobacillus plantarum* and *Streptococcus thermophilus* are psychobiotic bacteria and synthesize some neurotransmitters that play a role in the pathogenesis of depression. In this study, we aimed to investigate the therapeutic effects of Bactolac (*Lactobacillus plantarum* NBIMCC 8767  + *Streptococcus thermophilus* NBIMCC 8258) on chronic stress-induced depression in rats. Behavioral tests, including the sucrose preference test, elevated plus maze test, forced swim test, and three-chamber sociability test, were employed to assess depressive and anxiety-like behaviors. The expression level of the 5-HT1A, DRD1, ADRA-2A, GABA-A α1, CNR1, NR3C2, NOD1, NLRP3 and MC4R; BDNF levels, glial activity and intestinal permeability were determined in chronic stress-induced depression in rats. In conclusions, chronic stress decreased the expression levels of 5-HT1A, DRD1, ADRA-2A, GABA-A α1, CNR1, NR3C2, NOD1 and BDNF level; increased the expression levels of NLRP3 and MC4R, caused neurodegeneration and glial activity, ultimately led to depressive effects. Bactolac was effective in reducing depressive-like behaviors according to the results of behavioral tests. Bactolac treatment provided high neuronal survival rate increasing BDNF level, prevented the excessive release of pro-inflammatory cytokines by reducing the expression levels of NLRP3 and MC4R, therefore, prevented the excessive activation of the hypothalamus–pituitary–adrenal (HPA) axis and accordingly, reduced neurodegeneration and glial cell activation in depressed rats. We can suggest that Bactolac supplementation may be beneficial in coping with stress, alleviate the effects of chronic stress and help to protect mental health.

## Introduction

Stress is a natural response that activates neural, hormonal and behavioral processes in the body to maintain homeostasis under the influence of threatening physical, biological and psychological stimuli inside and outside our body [41, 55]. Chronic stress, which poses serious threats by not being able to adapt, occurs as a result of long-term/recurrent and intense exposure to stressors and continues for a long time. Chronic stress causes cellular, neurochemical and neuroanatomical changes that have damaging effects on brain functions and is a risk factor for mental health [10, 41, 103, 104]. According to the Trier Inventory, the factors that cause chronic stress are: chronic worrying, excessive workload, social overload, social isolation, performance pressure, work dissatisfaction, excessive demands from work, social conflicts and lack of social recognition. Chronic stress, which is very harmful to health, plays a role in the onset and development of depression, a common mood disorder, and causes negative effects such as persistent sadness and loss of interest in people with psychiatric illnesses, and also leads to frequent episodes of depression [25, 49, 60]. Because chronic stress increases people's emotional reactivity, it can precipitate depression in individuals who are sensitive to stressors [91, 107].

Depression, the most common psychiatric disease that causes devastating and debilitating effects on a person's mental and physical health, is a chronic mental illness affecting approximately 450 million people worldwide according to the WHO, it will constitute the biggest global burden by 2030 [84, 109]. According to the Diagnostic and Statistical Manual of Mental Disorders, 5 th edition (DSM-V) and the ICD- 10 Classification of Mental and Behavioral Disorders, some of the symptoms of depression which are twice as common in women than in men, are depressed mood, loss of pleasure and interest (anhedonia), anxiety, pessimism, changes in appetite or weight, headache and muscle pain, palpitation, fatigue, sleep disorders, and loss of sexual function. Other symptoms of depression include excessive guilt, low self-esteem, decreased motivation, psychomotor agitation or retardation, feelings of worthlessness, cognitive impairments, recurrent thoughts of death or suicide, and the two main symptoms (persistent depressed mood and anhedonia) plus at least four of the other symptoms must persist for two weeks or more [33, 48, 82, 90, 116]. Chronic stress is the main reason of mood disorders such as anxiety and depression [99]. Chronic stress is a factor that facilitates and accelerates the onset of depression, and can also lead to behavioral changes resembling clinical depression [115]. The hippocampus is the brain structure highly affected by stress, the stress-sensitive hippocampal region may contribute to the development of depressive disorder and play a central role in depression [16, 50]. Although there are numerous clinical and preclinical studies, the pathophysiology of depression has not been fully elucidated. This is because the factors that lead to depression are highly diverse. Despite the availability of several clinically effective treatments for depression, around 30%–40% of patients do not respond to antidepressant medications [13, 62, 94]. Serious side effects of antidepressant drugs or failure to achieve the desired remission cause general deterioration in health. These situations have led to the search for natural treatment methods that are safe in terms of side effects, and in this regard, it is important to develop biological therapeutic agents [78, 121].

Brain-derived neurotrophic factor (BDNF) is an important neurotrophic factor that provides neuronal survival and differentiation. It plays a role in modulating neural networks and provides protective effects for hippocampal neurons against oxidative, metabolic, and excitotoxic stress. Stress has been shown to affect the synthesis and release of BDNF in neurons. Numerous studies have suggested that depression is associated with reduced BDNF levels [4, 68]. The NLRP3 inflammasome is essential in the pathophysiology of stress-induced depression. Activation of the NLRP3 inflammasome has been observed in individuals with major depressive disorder, indicating that NLRP3 could serve as a potential novel therapeutic target for managing depressive disorders [3]. The melanocortin 4 receptor (MC4R) plays a significant role in regulating the activity of the hypothalamus–pituitary–adrenal (HPA) axis and is functionally and anatomically linked to corticotropin-releasing factor (CRF), a key mediator in stress and stress-related behaviors. MC4R exhibits widespread to moderate expression in structures of the limbic system, such as the hippocampus. There is also a correlation between the monoaminergic systems and the expression of MC4R. Given this relationship, MC4R may represent a potential therapeutic target for addressing stress-associated conditions such as anxiety and depression [18]. The hippocampus receives inputs from neurons that release serotonin, norepinephrine, and dopamine. Additionally, GABAergic projections innervate all regions of the hippocampus [4]. Both the monoamine systems and glucocorticoids have been involved in the modulation of neuronal plasticity and the process of neurogenesis in the hippocampus [24]. The endocannabinoid system plays a key role in modulating cognition, mood, emotional responses and stress regulation, primarily through the activation of cannabinoid receptor 1 (CB1/CNR1) [129]. Glucocorticoids and mineralocorticoid receptors (MRs) play a significant role in stress-induced hippocampal atrophy [4]. MR is highly expressed in limbic brain structures, including the hippocampus, where it plays a role in maintaining a basal inhibitory regulation of cortisol secretion. Individuals with depression show reduced expression of the MR in the hippocampus and prefrontal cortex. Furthermore, various polymorphisms and haplotypes of the MR gene (NR3 C2) have been linked to depression [119]. MR could potentially serve as a therapeutic target for treatment [105]. Elevated corticosteroid levels, along with stressful conditions, may be associated with accelerated damage and eventual loss of hippocampal pyramidal neurons, which are key sites for glucocorticoid activity. These observations suggest that stress-induced cognitive dysfunctions may result from the dysfunction or degeneration of hippocampal neurons [40]. Chronic stress and depression induce apoptosis, leading to neuronal loss in individuals with depression. The activation of caspase- 3 serves as a biomarker for neuronal apoptosis [7]. An increase in Ki- 67 immunopositivity serves as a marker of glial activation [79]. The activation of glial cells contributes to the pathophysiology of depression [126]. Activated microglia release pro-inflammatory cytokines, which contribute to the development of depressive symptoms [75]. Excessive microglial activation is involved in a range of brain disorders, including neurodegenerative diseases and depression. As the primary mediator of neuroinflammatory processes, microglial activation leads to neuroinflammation, which is a key factor in the development of depression. Inflammatory or stress-induced dysfunctions driven by microglia are frequently observed in depression [34, 53]. Chronic stress increases intestinal permeability, which is also observed in individuals with depression [67, 74]. The NOD1 receptor plays a crucial role in maintaining the normal function of the gastrointestinal tract and influences cognition, anxiety, serotonergic activity in both central and peripheral systems, as well as HPA axis activation. Expression of NOD1 receptors in intestinal epithelial cells modulates behavior. Inhibition of the NOD1 receptor leads to the onset of symptoms associated with stress-induced anxiety, cognitive dysfunction, and depression [87].

The gut microbiota impacts various essential processes in the human body, including nutrition, host defense, the regulation of immune reactions and psychological disorders. Due to its size and vital functions, the intestinal commensal microbiota is often referred to as a vital organ. Because of their large numbers and diversity, normal physiology can be significantly affected and depending on this, the host's susceptibility to diseases can accordingly be altered. Intestinal bacteria play multiple roles, including digestion, production of short-chain fatty acids, vitamin synthesis, maintenance of optimal immune system function, and influencing the permeability of the mucosal barrier. These bacteria significantly affect metabolism and central nervous system functions via the gut-brain-microbiome axis via neural, endocrine and immune mechanisms, thus affecting mental health. The change in intestinal microbiota in favor of bad bacteria is called dysbiosis and is commonly observed in depression and neurodegenerative disorders. Generally, the diversity of the gut microbiota is reduced in major depressive disorder. The microbiota–gut–brain axis is a bidirectional communication pathway between the gut microbiota and the brain. The vagus nerve, the 10 th and longest cranial nerve in our body, transmits comprehensive information from the gut to the brain (afferent pathway) and from the brain to the gut (efferent pathway). Afferent pathways provide modulation of the HPA axis, which regulates the secretion of stress-related hormones. The HPA axis plays a vital role in stress management in the body. Changes in vagal signaling may contribute to the development of depression, and stimulation of the vagus nerve may improve mood and provide beneficial effects. Some probiotics can stimulate the vagus nerve and produce antianxiolytic effects. Probiotics are live microorganisms that are beneficial to host health when consumed in sufficient quantities. Psychobiotics are probiotic bacteria that, when consumed in sufficient quantities, provide antidepressive effects in individuals with psychiatric disorders [23, 109]. *Lactobacillus plantarum* and *Streptococcus thermophilus* are psychobiotic bacteria and synthesize some neurotransmitters that play a role in the pathogenesis of depression [76].

Since antidepressant drugs have many serious side effects and cannot provide the desired level of treatment response in depression patients, natural alternative treatment methods are needed and in this study, we aimed to investigate the therapeutic effects of Bactolac (*Lactobacillus plantarum* NBIMCC 8767 + *Streptococcus thermophilus* NBIMCC 8258) on key receptors in the pathophysiology of stress and depression such as 5-HT1 A, DRD1, ADRA- 2 A, GABA-A α1, CNR1, NR3 C2, NOD1, NLRP3 and MC4R; BDNF levels, glial activity and intestinal permeability in chronic stress-induced depression in rats, additionally, we subjected the rats to four different behavioral tests to assess depressive-like behaviors.

## Materials and methods

### Experimental animals

Male Wistar Albino rats were purchased from Sakarya University Experimental Medical Applications and Research Center, the study was performed in this center. All procedures involving animals were complied the European Community Council Directive of 24 November 1986, and ethical approval was granted by the Sakarya University Ethics Committee (SAU HADYEK, Number:02/11/2022–42, Sakarya, Türkiye). All experimental animals were housed in polycarbonate transparent cages and maintained under standard laboratory conditions (12/12 light/dark cycle, temperature (22 ± 2 °C), and humidity (45–50%)), fed standard pellet feed and provided with tap water.

### Preparation of Bactolac

Bactolac which contains live bacteria in liquid form and stored at + 4 °C, was taken into test tubes under sterile conditions and then the test tubes were placed in the hot air oven and kept at + 37 °C for 10 min (BM CO Food Agriculture Tourism Industry and Trade Incorporated Company provided Bactolac).

### Experimental design

**Fig. 1:**
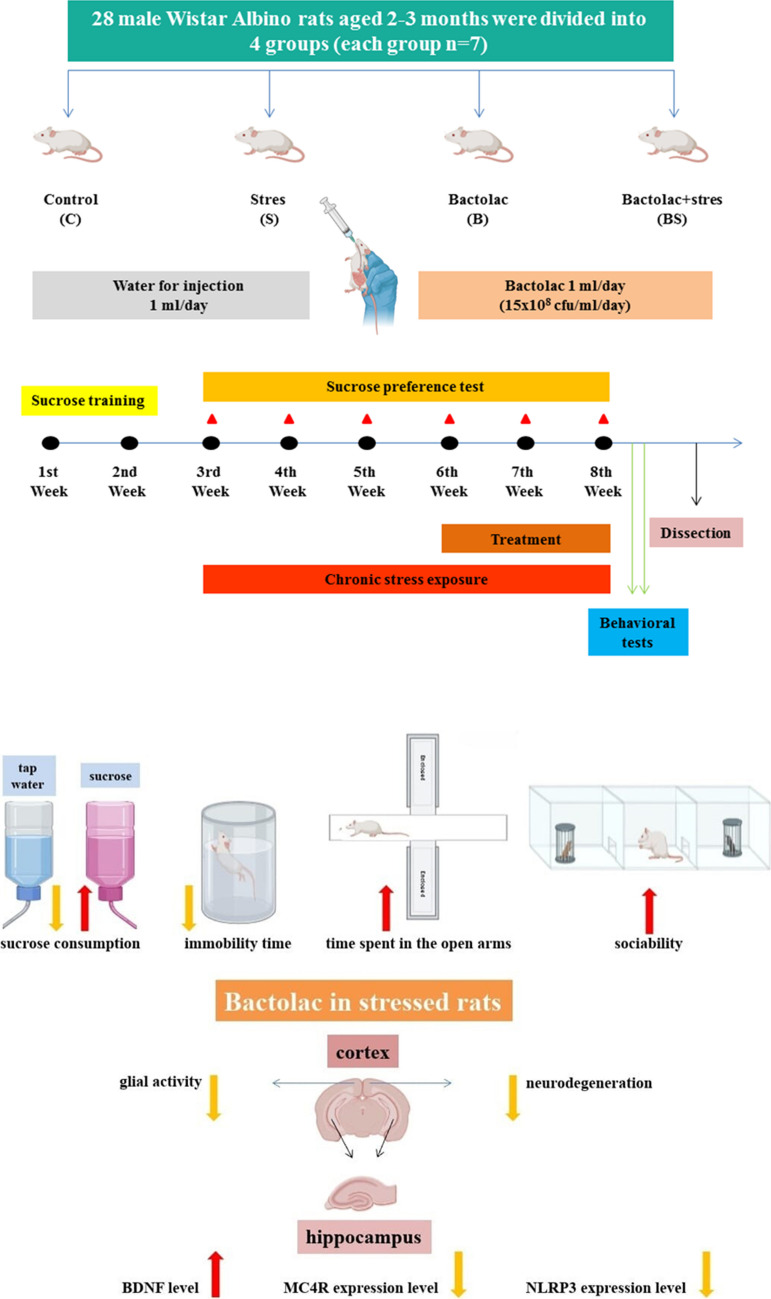
Schematic representation of the experimental design and antidepressant effects of Bactolac in depressed rats. (Made in BioRender)

The rats were randomly divided into four different groups (each *n* = 7): Control group (C), stress group (S), Bactolac group (B), Bactolac + stress group (BS). The C and S groups received 1 ml/day of water for injection by gavage, the B and BS groups received 1 ml/day of Bactolac (15 × 10^8^ cfu ml/day) by gavage. Chronic stress protocol was performed to the S and BS groups, and the rats in these groups were kept in different rooms. The experimental design was shown in Fig. 1, (Table 1). This study was performed simultaneously with another study (ethics numbered SAU HADYEK 12/01/2022–07, supported by project No:2022–9–32–13, Sakarya University Scientific Research Projects Unit) on chronic stress-induced depression. Data from rats in the control and stress groups in both studies were used in accordance with the 3Rs (Replacement, Reduction and Refinement).

**Table 1:** Experimental Groups

Groups	Administered substance	Dose	Volume	Method	Duration
Control (C)	Water for injection	1 ml/day	1 ml	Gavage	21 days
Stress (S)	Water for injection	1 ml/day	1 ml	Gavage	21 days
Bactolac (B)	Bactolac	15 × 10^8^ cfu mL/day	1 ml	Gavage	21 days
Bactolac + Stress (BS)	Bactolac	15 × 10^8^ cfu mL/day	1 ml	Gavage	21 days

### Chronic unpredictable mild stress (CUMS) protocol

Because of long-term exposure to stressful events in life can cause depression, the CUMS model, which is the most common and frequently used experimental depression model, is used to examine depressive-like behaviors, depression, and various treatments [73, 83]. CUMS procedure performed to the rats according to the Willner protocol (modified) [29, 56, 117, 118], (Table 2). Rats in the S and BS groups were exposed to chronic stress for six weeks starting from the third week. To prevent rats from adapting to the stressors, each stressor was applied on a different day each week. It was determined by the sucrose preference test whether the rats were stressed or not, and Bactolac treatment started in the sixth week according to the test results.

**Table 2:** Chronic stress protocol

Stressor	Duration
45° cage tilt	twice a week, 21–24 h
Wet sawdust	once a week, 21 h
Loud noise (85–90 dB)	3 times a week, 3 h
Overnight illumination	twice a week
Immobilization at + 4 °C	once a week, 3 h
Food and water deprivation	once a week, 21–24 h
Restricted access to food (5 pellets)	once a week, 1 h
Bright flashing light (300 times/min)	3 times a week, 3 h
Leaving alone in dark cage sized 12 × 12x12 cm^3^ (daytime)	once a week, 1–2 h
Changing the cage (coming together with stranger partners)	once a week, 2–3 h

### Behavioral tests

Four different behavioral tests were performed to evaluate depressive behaviors. Sucrose preference test was performed once a week starting from the 3rd week, other behavioral tests were performed at the end of the eighth week.

### Sucrose preference test (SPT)

The SPT was modified and based on previous study [62, 100]: Before starting the sucrose preference test, all experimental animals were subjected to sucrose water training for 2 weeks. 500 ml of 1% sucrose water was prepared. On the first day, 2 bottles containing sucrosed water were placed in the cages. On the 2nd day, a sucrosed water bottle was replaced with a bottle containing 500 ml tap water. On the 3rd day, feed and water bottles were removed for 24 h. On the 4 th day, 1 sucrosed water bottle and 1 tap water bottle were placed in the cages, and sucrosed water consumption was calculated at the end of the 1-h period. To test sucrose preference for 6 weeks, two 200 ml water bottles, one containing tap water and the other containing 1% sucrose, were placed in cages for 1 h. Sucrose water consumption of rats deprived of food and water for 24 h was calculated according to the following formula at the end of 1 h: Sucrose preference = [sucrose consumption/(sucrose consumption + tap water consumption)] × 100. If sucrose preference is below 65%, rats are considered to show anhedonia.

### Three-chamber sociability test (Social interaction test) (TCST)

The test was performed as previous study with some modifications [124]: For the test, a cage made of transparent durable plastic material size 40 × 35x60 cm and divided into three equal chambers (size 40 × 35x20 cm) with two transparent plexiglass plates size 40 × 35 cm was used. In order for the rats to pass between the chambers, size 5 × 5 cm passage holes were made in the middle of the bottom of the plexiglass plates [51, 58]. Each rat was first placed in the center, and at the same time, a stranger male rat was placed in the cage in the left chamber of the apparatus. After 150 s, a second stranger male rat was placed in the cage in the right chamber. Time the rat spent in the chambers, its interaction with unfamiliar rats, and the rearing numbers were recorded for 5 min.

### Elevated plus maze test (EPMT)

The EPMT was modified and based on previous study [106]: The black coloured, plus-maze-shaped test apparatus was made of glass + metal mixture material, consisting of two open arms size 10 × 110 cm and two closed arms size 10 × 40x50 cm, and having a height of 50 cm from the ground. Experimental animals were placed in the center of the apparatus, facing the open arm, and the time spent in the open arms and closed arms was recorded for 5 min. Open and closed arms were cleaned with 10% alcohol and dried after each rat.

### Forced swim test (FST)

The FST performed as described in previously [17]: The test consists of two sessions. In both sessions, clean water at a temperature of 24–26 °C was used and when the water became dirty, it was replaced with clean water. The first session, which lasted 15 min, was performed to acclimate the rats to the test environment. The first session was performed before treatment was administered and without behavioral recording. After 24 h, in the second session lasted 5 min, the time the rats remained completely motionless was recorded.

### Collecting tissue samples

After the behavioral tests performed at the end of the eighth week, the rats were sacrificed by intracardiac blood collection under ketamine (80 mg/kg)-xylazine (5 mg/kg) anesthesia. The brain tissues were quickly dissected, and the right and left cerebral hemispheres were separated, hippocampal tissues collected from both hemispheres. The ileum portion of the small intestine was collected and stored at − 80 °C until examined. Also, brain tissues and intestinal tissues placed in histology cassettes were fixed in 10% buffered neutral formaldehyde solution for histopathological examinations.

### Determination of BDNF level

BDNF (brain-derived neurotrophic factor) levels in the left hippocampus tissue were determined using the ELK Biotechnology Rat BDNF Elisa Kit (ELK5459) in accordance with the manufacturer's instructions.

### Determination of 5-HT1 A, DRD1, ADRA- 2 A, GABA-Α α1, CNR1, MC4R, NR3 C2, NLRP3 receptor expression levels in hippocampus tissue and NOD1 receptor expression levels in small intestine tissue by RT-PCR

The expression level of the genes planned to be studied in the right hippocampus and small intestine tissues were determined using the Promega A6001 RT PCR kit, in accordance with the manufacturer's instructions. Primers were shown in Table 3.

**Table 3:** Primers

Gene	Prımer	Sequence
5-HT1 A (388 bp)	Forward	5'-CCAAAGAGCACCTTCCTCTG- 3'
	Reverse	5'-TACCACCACCATCATCATCA- 3'
DRD1 (108 bp)	Forward	5′-GACACAAGGTTGAGCA- 3′
	Reverse	5′-CTGGGCAATCCTGTAGATA- 3′
ADRA2a (112 bp)	Forward	5′-TTCTTTTTCACCTACACGCTCA- 3′
	Reverse	5′-TGTAGATAACAGGGTTCAGCGA- 3′
GABA-A α1 (304 bp)	Forward	5′-AGCTATACCCCTAACTTAGCCAGG- 3′
	Reverse	5′-AGAAAGCGATTCTCAGTGCAGAGG- 3′
CNR1 (306 bp)	Forward	5′-ATGAAGTCGATCCTAGATGGCCTTG- 3′
	Reverse	5′-GTTCTCCCCACACTGGATG- 3′
MC4R (431 bp)	Forward	5′-AGTCTCTGGGGAAGGGGCA- 3′
	Reverse	5′-CAACTGATGATGATCCCGAC- 3′
NR3 C2 (260 bp)	Forward	5′-GCTCAACATTGTCCAGTACA- 3′
	Reverse	5′-GCACAGGTGGTCCTAAGATT- 3′
NLRP3 (313 bp)	Forward	5′-TCTGTTCATTGGCTGCGGAT- 3′
	Reverse	5′-GCCTTTTTCGAACTTGCCGT- 3′
NOD1 (149 bp)	Forward	5′-TAGCCTTCTGCAATGCTTGTTC- 3′
	Reverse	5′-CCGTGAGACGGCTAAAGCAA- 3′
β-actin (150 bp)	Forward	5′-CCTGTGGCATCCATGAAACTAC- 3′
	Reverse	5′-CCAGGGCAGTAATCTCCTTCTG- 3′

### Histopathological analysis in the cortex and small intestine tissues and immunohistochemical staining

Histopathological examinations were performed according to previous studies [28, 72, 86, 110]. All tissue samples were placed in 10% buffered neutral formaldehyde solution and kept for 48 h for tissue fixation. Tissue samples, which fixation procedures were completed, were embedded in paraffin. Sections of 4 µm thickness were placed on slides for H-E staining and after the H-E staining process was completed, the slides were examined under the light microscope. To assess tissue damage, Caspase- 3 (Caspase- 3 Antibody, Genetex) and Ki- 67 (Ki- 67 Antibody, Genetex) antibodies were used to detect cell proliferation. Paraffin-embedded tissue sections of 4–5 microns were placed in polylysine coated slides. The slides were placed in an oven at 56 ºC for 1 h for thermal treatment to remove the paraffin. The sections were then processed through the following steps: 2 × 10 min in toluene, 2 × 5 min in absolute alcohol, 2 × 5 min in 96% alcohol, 2 × 5 min in 90% alcohol, 2 × 5 min in 70% alcohol, and 1 × 5 min in distilled water. The sections were incubated in 0.5% H_2_O_2_ in methanol for 10 min in the dark to block endogenous peroxidase activity, followed by two washes in PBS. To antigen retrieval, the sections were placed in a microwave oven with 10 mM citrate buffer (pH = 6) and heated in a microwave oven for two 10-min cycles. The sections taken out from microwave oven were left to cool at room temperature for 20 min and washed twice with PBS. For immunohistochemistry, firstly, Ultra V Block solution (LabVision, UltraVision Large Volume Detection System Anti-rabbit, HRP) was applied to the sections for 30 min and then primary antibody (Rabbit Caspase- 3 and Ki- 67, Neomarkers) diluted at 1/200 ratio was applied to the sections for 30 min, followed by two PBS washes. The sections were then incubated with Biotinylated Goat Anti-Rabbit secondary antibody (LabVision, UltraVision Large Volume Detection System Anti-rabbit, HRP) for 30 min, followed by two PBS washes. Streptavidin Peroxidase (LabVision, UltraVision Large Volume Detection System Anti-rabbit, HRP) was applied for 30 min, followed by two PBS washes. In the dark, the tissues were stained with the chromogen (LabVision AEC Substrate System) for 20 min (20 ml AEC chromogen + 1 ml AEC substrate thoroughly mixed). After washing with distilled water, the sections were counterstained with hematoxylin for 10 min, then left in tap water for 7 min. After the preparations were dried, they were covered with sealer (Aqueous Mounting Medium, ScyTek). Caspase- 3 and Ki- 67 staining percentages were determined using the H-Score method. The staining intensity was scored in five randomly selected areas, and the area with the highest score was identified. In both groups, at least 200 cells were marked in each 40 × magnification field. The percentage of stained cells and the staining intensity were used as criteria for evaluation. H Score = (3xpercentage of strongly staining nucleus) + (2xpercentage of moderately staining nucleus) + (1xpercentage of weakly staining nucleus).

### Statistical analysis

Analyzes were made using the GraphPad Prism 8 program. Because the groups exhibited normal distribution, one-way analysis of variance (One-way ANOVA) was used for comparison between groups. In multiple comparisons, Dunnet's test was used to compare the control group with other groups while Tukey's HSD test was used to compare other groups except the control group. By taking the statistical significance level as 0.05, the statistical significance of the data in the present study was expressed as *p < 0.05, **p < 0.01 and ***p < 0.001.

## Results

### Evaluation of the BDNF levels

BDNF levels decreased in the S group compared with the C, B and BS groups. Statistically significant difference was found between the C-S, S-B groups (F:5.379; *p < 0.05), (Fig. 3).

### Evaluation of the behavioral tests

**Fig. 2:**
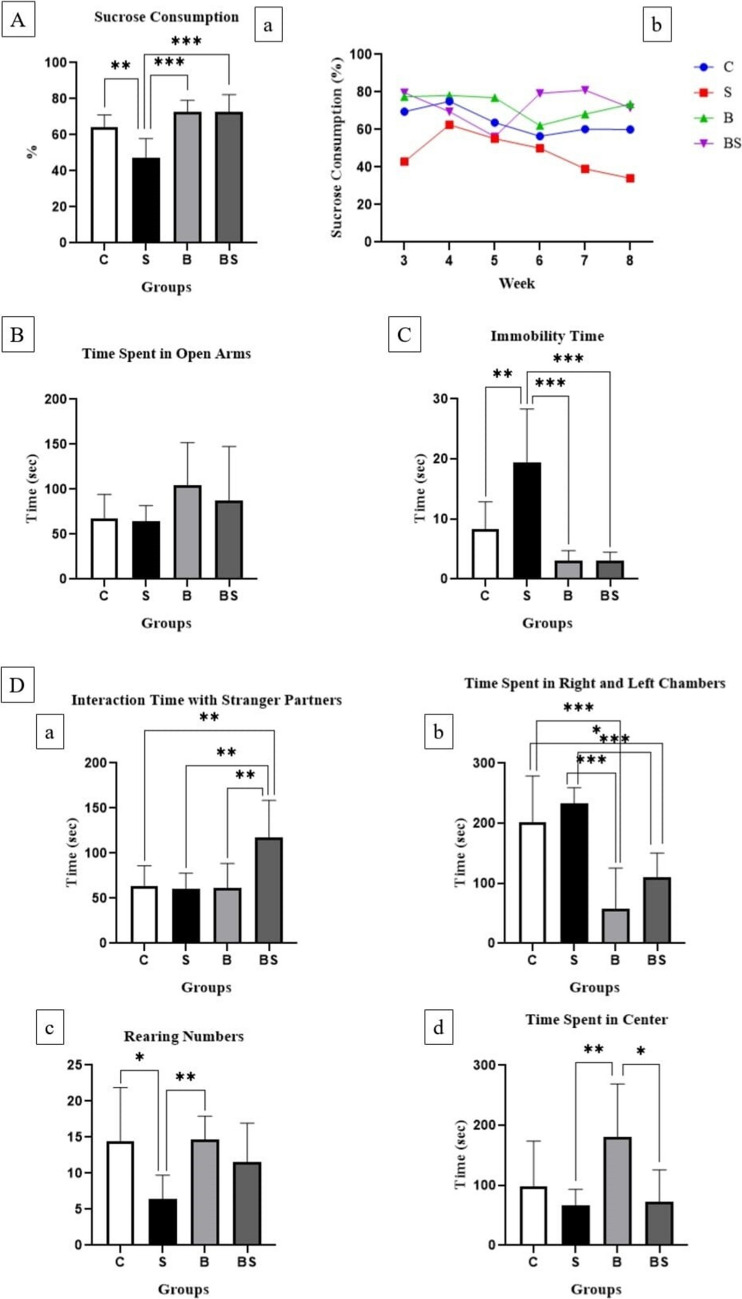
**A**) Sucrose preference test: (**a**) Percentage of sucrose consumption rate (%) and (**b**) weekly change in sucrose consumption (%),(F:12.03; ***p < 0.001) **B**) Elevated plus maze test: Time spent in open arms (F:1.439; *p* > 0.05) **C**) Forced swim test (FST): Immobility time (F:15.91; ***p < 0.001) **D**) Three-chamber sociability test (social interaction test): (**a**) interaction time with stranger partners (F:6.511; **p < 0.01), (**b**) time spent in right and left chambers (F:14.46; ***p < 0.001), (**c**) rearing numbers (F:3.910; *p < 0.05), (**d**) time spent in center (F:4.540; *p < 0.05). Control group (C), stress group (S), Bactolac group (B), Bactolac + stress group (BS)

The sucrose consumption results were determined based on weekly consumption rates (%) from the 3rd week onwards over the 6-week period. The sucrose consumption results were determined based on weekly consumption rates over the 6-week period. From the 4 th week onwards, sucrose consumption decreased continuously in the S group due to the effect of chronic stress, and it increased in the B and BS groups. Statistically significant difference was found between the C-S, S-B, S-BS groups (F:12.03; ***p < 0.001), (Fig. 2). The time spent in the open arms decreased in the S group due to chronic stress, Bactolac increased the time spent in the open arms in the B and BS groups. There was no statistically significant difference between the groups (F:1.439; *p* > 0.05), (Fig. 2). The immobility time increased in the S group, Bactolac decreased the immobility time in the B and BS groups Statistically significant difference was found between the C-S, S-B, S-BS groups (F:15.91; ***p < 0.001), (Fig. 2). Social interaction times were similar in the C, S and B groups. The rearing numbers were less in the S group compared to the C group, similar rearing numbers were seen in the B and C groups. The time spent in the center was similar in the S and BS groups. The time spent in the right and left chambers was higher in the C and S groups, but this occurred when there was no unfamiliar partner in a chamber, meaning it was not due to social interaction. Statistically significant difference was found between the groups for the interaction time, rearing numbers, time spent in the center, right and left chambers respectively; C-BS, S-BS, B-BS (F:6.511; **p < 0.01); C-S, S-B (F:3.910; *p < 0.05); S-B, B-BS (F:4.540; *p < 0.05); C-B, C-BS, S-B, S-BS (F:14.46; ***p < 0.001), (Fig. 2).

### Evaluation of the gene expression levels

5-HT1 A, DRD1, ADRA- 2 A, GABA-A α1, CNR1, MC4R, NR3 C2, NLRP3 receptor expression levels in the right hippocampus tissue; NOD1 receptor expression levels in small intestine tissue were determined by RT-PCR method.

**Fig. 3:**
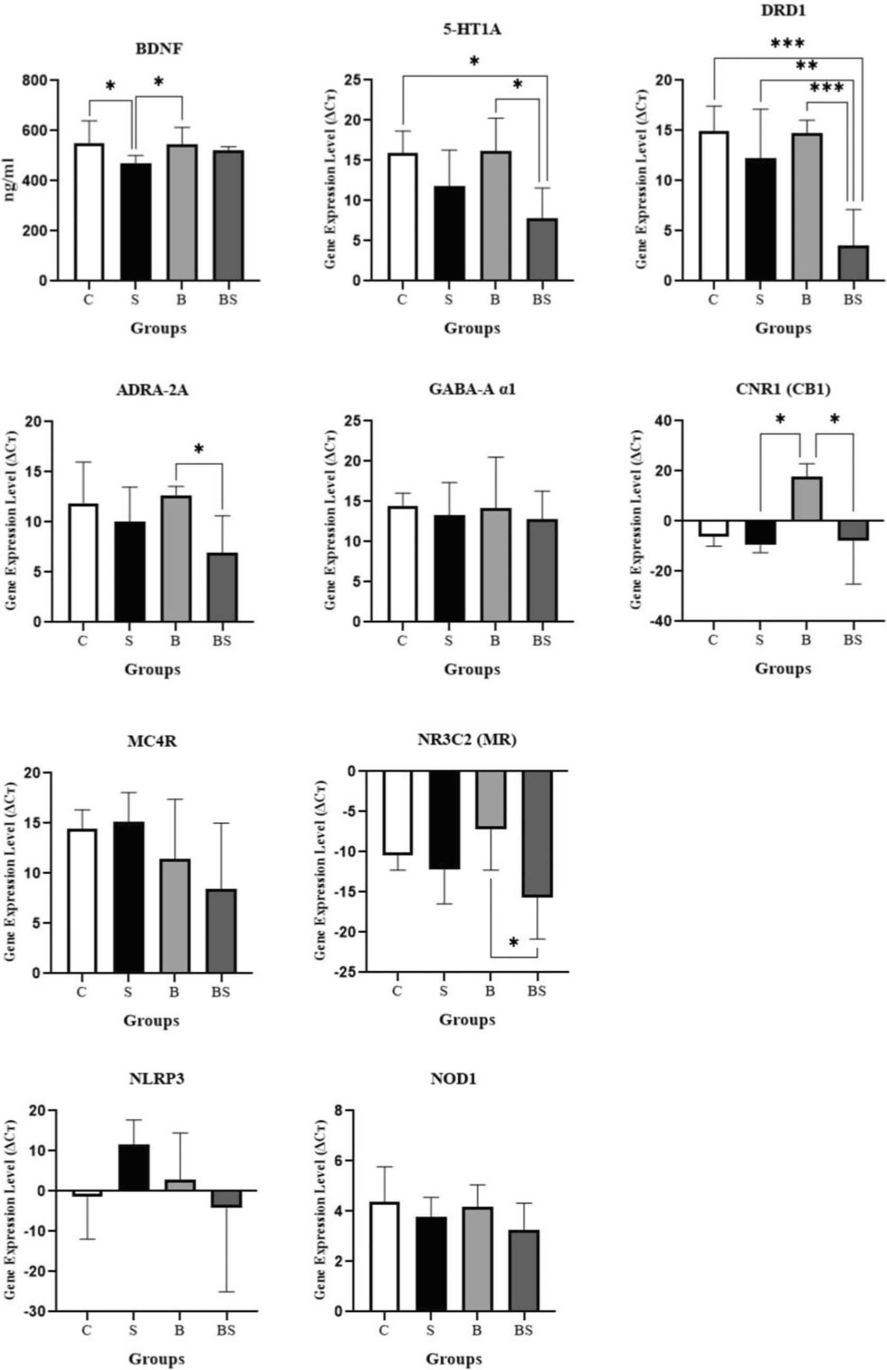
BDNF levels (F:5.379; *p < 0.05) and 5-HT1 A (F:5.318; **p < 0.01), DRD1 (F:13.10; ***p < 0.001), ADRA- 2 A (F: 4.640; *p < 0.05), GABA-Α α1 (F:0.1803; *p* > 0.05), CNR1 (*p < 0.05), MC4R (F:2.070; *p* > 0.05), NR3 C2 (F:3.368; *p < 0.05), NLRP3 (F:1.315; *p* > 0.05) receptor expression levels in hippocampus tissue and NOD1 receptor expression levels (F:1.143; *p* > 0.05) in small intestine tissue. Control group (C), stress group (S), Bactolac group (B), Bactolac + stress group (BS)

5-HT1 A, DRD1 and ADRA- 2 A expression levels decreased due to chronic stress in the stress group compared to the control group. Statistically significant difference was found between the groups respectively; C-BS, B-BS (F:5.318; **p < 0.01); C-BS, S-BS, B-BS (F:13.10; ***p < 0.001); B-BS (F:4.640; *p < 0.05), (Fig. 3). GABA-A α1 expression level decreased in the S group. Bactolac was not effective in increasing GABA-A α1 expression levels in stressed rats, the results in the B and C groups were similar. There was no statistically significant difference between the groups (F:0.1803; *p* > 0.05), (Fig. 3). NLRP3 expression level was significantly higher in stressed rats. Bactolac treatment was effective in reducing NLRP3 expression level in stressed rats. There was no statistically significant difference between the groups (F:1.315; *p* > 0.05), (Fig. 3). CNR1 expression was downregulated in the C, S and BS groups. Bactolac was effective in increasing CNR1 expression in the B group. Statistically significant difference was found between the S-B, B-BS groups (*p < 0.05), (Fig. 3).

MC4R expression level increased in the stress group. There was no statistically significant difference between the groups (F:2.070; *p* > 0.05), (Fig. 3). We found MR expression level to be downregulated in all groups. Statistically significant difference was found between the B-BS groups (F:3.368; *p < 0.05), (Fig. 3). NOD1 expression level decreased in the S group, Bactolac was not effective in increasing NOD1 expression level in stressed rats, but NOD1 expression level was similar in the C and B groups. There was no statistically significant difference between the groups (F:1.143; *p* > 0.05), (Fig. 3).

### Evaluation of the histopathological examinations

**Fig. 4:**
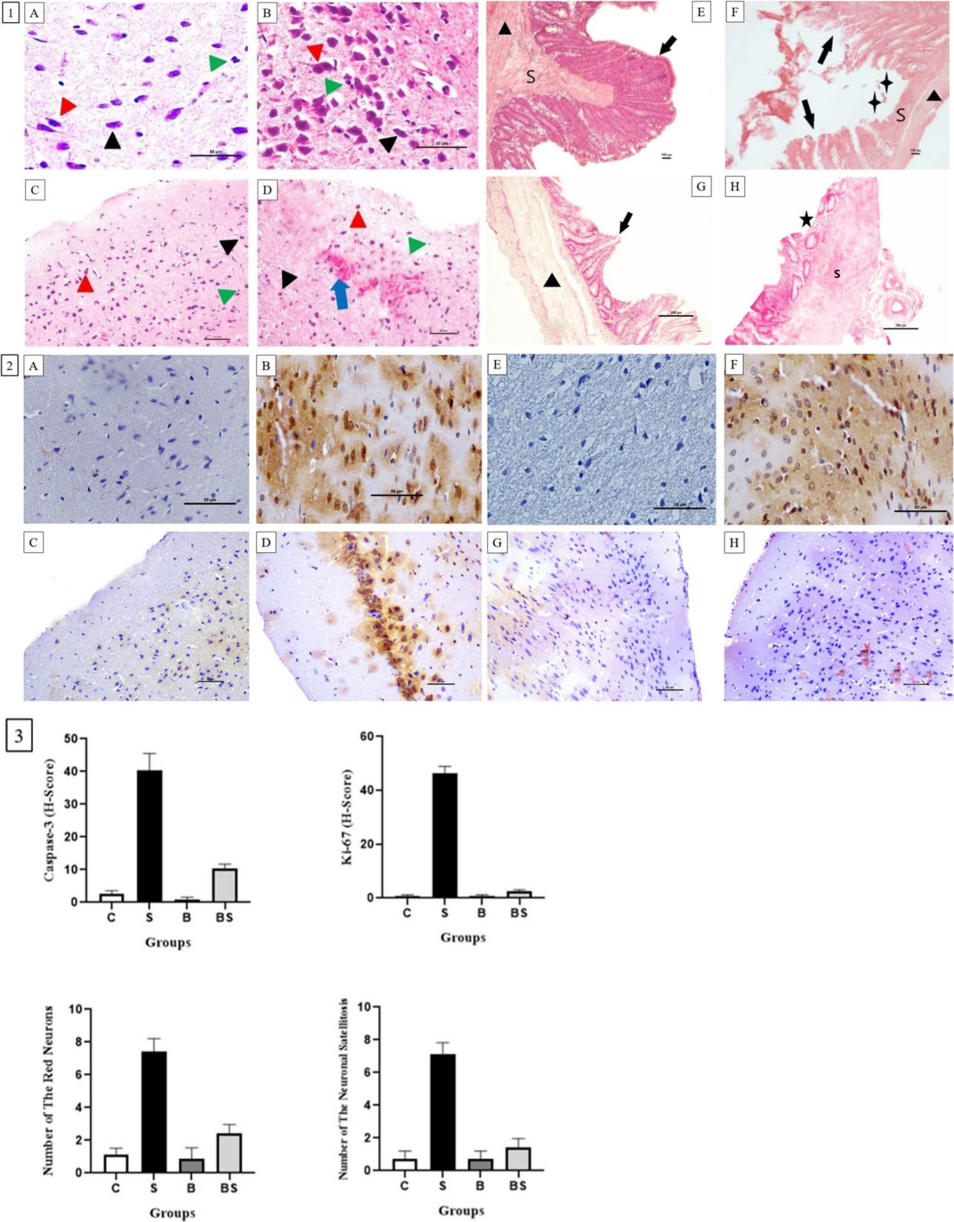
1. Histological evaluation of cortex tissue: red neurons and neuronal satellitosis: Control group (**A**), stress group (**B**), Bactolac group (**C**), Bactolac + stress group (**D**), (bar: 50.0 μm, 20X, HE). Black arrows show normal neurons, red arrows show damaged neurons, blue arrow shows bleeding and green arrows show increased number of neuroglial cells (neuronal satellitosis). Histological evaluation of small intestine tissue: intestinal permeability: Control group (**E**), stress group (**F**), Bactolac group (**G**), Bactolac + stress group (**H**), (bar: 100.0 μm, 20X, HE). 2. Caspase- 3 activity in cortex tissue: control group (**A**), stress group (**B**), Bactolac group (**C**), Bactolac + stress group (**D**), Ki- 67 activity in cortex tissue: Control group (**E**), stress group (**F**), Bactolac group (**G**), Bactolac + stress group (**H**), (bar: 50.0 μm, 40X), 3. Graphical representation of the histopathology results: (a) number of the red neurons, (b) number of the neuronal satellitosis, (c) Caspase- 3 (H-Score), (d) Ki- 67 (H-Score). **S**: Submucosa,  : Muscularis layer,  : Villus layer,  : Ulceration area

The cells stained brown due to the use of a chromogen indicate the levels of Caspase- 3 and Ki- 67 activity. The number of red neurons and Caspase- 3 activity, which are markers of neurodegeneration, as well as the number of neuronal satellitosis, were found to be significantly greater in the S group than the C group. A similar number of red neurons and neuronal satellitosis were observed in the B and C groups. Bactolac treatment was effective in reducing the number of red neurons and neuronal satellitosis in the BS group, and reduced the level of neurodegeneration that increased due to the effect of stress. Ki- 67 activity (glial activity) was significantly high in stressed rats. A similar level of Ki- 67 activity was seen in the B and C groups, and Ki- 67 activity was significantly reduced in stressed rats treated with Bactolac, (Fig. 4). Villus, submucosa and muscularis layers preserved structure were observed in the B group. Ulceration areas were seen in the BS group, and microvillus layers were not high, (Fig. 4).

## Discussion

Neuropsychiatric treatments have many different and serious side effects in the long term, and therefore, based on the bidirectional gut-brain axis, in this study, we investigated the therapeutic effects of Bactolac in chronic stress-induced depression in Wistar Albino rats. 5-HT1 A, DRD1, ADRA- 2 A, GABA-A α1, CNR1, MC4R, NR3 C2, NLRP3 receptor expression levels in hippocampus tissue; NOD1 receptor expression levels in small intestine tissue were determined by RT-PCR method. BDNF levels in hippocampus tissue were determined by elisa kit. Histopathological examinations were performed on cortex and small intestine tissues. To evaluate depressive behaviors, four different behavioral tests were performed: sucrose preference test, three-chamber sociability test (social interaction test), elevated plus maze test, and forced swim test.

The SPT is performed to determine the level of anhedonia, one of the two main symptoms of depression [61]. *Lactobacillus plantarum* DP189 increased sucrose consumption which decreased due to chronic stress [125]. In our study, Bactolac was effective in increasing sucrose consumption, reduced anhedonia in stressed rats. The EPMT is performed to evaluate risk taking/anxiety-related behaviors [106]. One study reported that the time spent in open arms by rats exposed to chronic stress decreased [69]. *Lactobacillus plantarum* PS128 treatment reduced anxiety-like behaviors [64]. *Lactobacillus plantarum* 286 and *Lactobacillus plantarum* 12407 increased the time spent in open arms and caused an anxiolytic effect [8, 99]. In this study, Bactolac treatment was effective in increasing the time spent in the open arms in stressful and non-stressful situation. The FST is performed to evaluate depressive-like behavior [108]. *Lactobacillus plantarum* DP189 reduced the increased immobility time caused by chronic stress [125]. A significant reduction in immobility time was seen in animals treated with *Lactobacillus plantarum* 286 [8]. In the present study, Bactolac reduced depressive-like behaviors in stressful situation. The TCST is performed to evaluate impairments in social interaction. *Lactobacillus plantarum* ST treatment increased social interaction time [124], a certain decrease in social interaction is indicative of an anxiogenic effect [36]. Interaction time with stranger partners decreased in rats exposed to chronic stress [92]. In the current study, Bactolac increased sociability in stressful condition.

5-HT1 A heteroreceptors, which mediate the physiological functions of serotonin in cognition, fear, anxiety and stress, are abundantly expressed in the prefrontal cortex, amygdala and hippocampus in the brain [2]. 5-HT1 A receptor, which plays an important role in the pathology of depression, has a critical role in the therapeutic effects of antidepressant drugs [37, 44]. DRD1 has an important function in the survival of newborn cells in the adult hippocampus, hippocampus-dependent synaptic plasticity and modulation of memory [45, 123]. α2 A receptors, which are important in noradrenergic transmission and expressed in both serotonergic and noradrenergic brain regions, play a role in the release of serotonin and norepinephrine. Since α2 A receptors are effective on mood and motivation, they are therapeutic targets for antidepressant and anxiolytic drug development. It was found that 5-HT1 A expression in the hippocampus decreased in rats exposed to chronic stress [111]. Stress-induced DRD1 downregulation was observed in the hippocampus in mice subjected to chronic mild stress [122]. The gene related to ADRA- 2 A was found to be downregulated in depressed male mice [42]. *Lactobacillus plantarum* PS128 increased serotonin and dopamine levels in the hippocampus in rats exposed to early life stress [65]. *Lactobacillus plantarum* treatment significantly reduced anxiety-related behaviors and modulated serotonergic signaling in the brain [26]. *Lactobacillus plantarum* DSM 24730 and *Streptococcus salivarius subspecies thermophilus* DSM 24731 probiotic mix increased DRD1 in the hippocampus, but was not very effective in increasing 5-HT1 A [11]. Levels of serotonin, norepinephrine, and dopamine in the hippocampus of mice increased after treatment with *Streptococcus thermophilus, Lactobacillus bulgaricus, Lactobacillus plantarum* and *Lactobacillus brevis* J1 [120]. In this study, Bactolac increased 5-HT1 A, DRD1 and ADRA- 2 A expression levels (monoamine levels) in non-stressful situations, however, it was not effective in stressful situation.

The GABA-A receptor plays a role in psychiatric and neurological disorders such as anxiety and depression, and the GABA-A receptor is a primary regulator of cognitive function and affect [70, 102]. GABA-A α1 receptor expression level decreased in the prefrontal cortex and hippocampus of rats subjected to restraint stress [128]. *Lactobacillus plantarum* increased GABA-A α1 expression levels in the brain of adult zebrafish [26]. Oral supplementation of *Lactobacillus plantarum* SNK12 increased mRNA levels of hippocampal GABA-A α1 in mice with chronic mild social defeat stress [108]. In our study, Bactolac was not effective in increasing GABA-A α1 expression levels in stressful condition.

NLRP3, a cytosolic receptor protein, recognizes danger signals reaching immune system cells such as macrophages and microglia and initiates inflammatory responses mediated by IL- 1β and IL- 18. Excessive release of pro-inflammatory cytokines IL- 1β and IL- 18, lead to activation of the HPA axis, which have harmful effects on brain structure and function and causes chronic neuroinflammation. Since NLRP3 signaling is involved in various neurological disorders and the NLRP3 inflammasome is active in depressed patients, the NLRP3 inflammasome is a novel therapeutic target for the treatment of stress-induced depression [3, 59, 93]. Compared to the chronic stress group, the hippocampal NLRP3 expression levels reduced in the group treated with *Lactiplantibacillus plantarum* CR12 [71]. *Streptococcus thermophilus* and *Lactobacillus plantarum* probiotic mix reduced NLRP3 levels in stressed rats [6]. In the present study, under stressful condition, Bactolac prevented excessive activation of HPA by reducing NLRP3 expression levels, possibly causing an immunosuppressive effect and thus reduced the release of pro-inflammatory cytokines, caused antidepressant effect.

BDNF which has an excitatory neurotransmitter function and is the most abundant neurotrophin in the brain, plays a role in neuronal development, increasing the survival and differentiation of neuron populations, synaptic plasticity, growth of dendrites and increasing dendrite density, neurotransmitter and neuropeptide production and excitability, learning, memory and behavior. BDNF has an important function in the adaptation processes required in depression and has both long- and short-term effects [12, 57, 68]. Chronic stress reduces the expression of BDNF, which has an important function in both the pathophysiology of the stress response and the pathogenesis of stress-related mood disorders. BDNF levels are reduced in patients with depression. Serum BDNF levels are evaluated in the treatment of depression [33, 57, 96]. BDNF is a potential therapeutic agent for degenerative disorders in the central nervous system [9]. In rats with CUMS induced depression, hippocampal BDNF levels decreased in the stress group [81]. In stressed mice, *Lactobacillus plantarum* SNK12 increased hippocampal BDNF level [108]. *Lactobacillus plantarum* IS- 10506 treatment increased brain BDNF expression in the hippocampus [54]. *Lactobacillus plantarum* ATCC 8014 increased brain BDNF levels in the hippocampus [89]. In the current study, Bactolac provided neuroprotective and antidepressant effect in stressful condition.

CB1 is the most abundant cannabinoid receptor in the brain; It is expressed in neurons, glial cells, gabaergic, glutamatergic, serotonergic and noradrenergic neuronal terminals, as well as in the main brain structures involved in stress-related behaviors such as the cerebral cortex, limbic system and hippocampus, and plays a role in the control of the HPA axis. Blockade of the CB1 receptor leads to loss of neurons in the hippocampus and inadequate levels of neurogenesis, excessive and long-term activation of the HPA axis by stress, increased sensitivity to neurotoxic conditions, and neuronal plasticity disorders [77, 98]. Pharmacological blockade of the endocannabinoid system is a risk factor in the pathogenesis of depression and anxiety disorders [5]. The endocannabinoid system regulates emotion, memory, cognition and stress responses, mood and depression through CB1 receptor activation. CB1 receptor signaling is vital for maintaining appropriate stress responses and emotional homeostasis in chronic stress exposure [31, 129]. *Lactobacillus plantarum* WJL treatment restored the hippocampal 2-AG level, which decreased due to stress [46]. In our study, Bactolac was effective in increasing CNR1 expression levels in non-stressful condition.

The MC4R, which is abundantly expressed in brain areas that regulate stress responses, plays a key role in this process. The melanocortinergic neural pathway is implicated in stress and stress-related psychiatric disorders, such as anxiety and depression. MC4R signaling has been demonstrated to trigger depressive-like behaviors and alter the neuronal activity of 5-HT neurons [15, 21]. The MC4R is associated with reduced social interaction and significantly affects the activity of the HPA axis [18]. MC4R in the hippocampus plays a critical role in regulating structural and functional plasticity. MC4R is expressed in microglia and astrocytes in addition to neurons [30, 38]. Blocking MC4R may be useful in treating patients with stress-related disorders such as anxiety and depression, and MC4R is a possible therapeutic target [18]. Chronic stress significantly increased the expression of MC4R in rats [97]. In this study, Bactolac provided a reduction in MC4R expression levels in stressful and non-stressful situation, led to an antidepressant effect.

Irregularity in the function of the HPA axis, which is an important part of the stress response system, is one of the most important mechanisms that lead to depression. Increased circulating glucocorticoid (cortisol) levels increase sensitivity to stressors and depressed mood, and impair memory and other cognitive functions [43, 63]. Limbic mineralocorticoid receptor (MR) is reduced during chronic stress and depression. Increased MR activity causes inhibition of HPA axis activity and also promotes slow-wave sleep; reduces anxiety, and changes circuitry to support coping. Therefore, MR activation may offer a target to alleviate depression and cognitive dysfunction [95]. Hippocampal MR expression decreased in rats subjected to long-term stress [27]. *Lactobacillus plantarum* PS128 decreased the expression of MR in the amygdala of rats [127]. In the present study, Bactolac was not found to be effective in increasing MR expression levels under stressful situation.

NOD1 promotes improvement of intestinal homeostasis and epithelial barrier function by modulating antimicrobial peptides, pro-inflammatory cytokines, autophagy, and adaptive immunity [66]. NOD1 is important for maintaining physiological function of the gastrointestinal tract, regulating central and peripheral serotonergic biology, cognition, anxiety, and HPA axis activation. Intestinal epithelial cell expression of NOD1 receptors regulates behavior. Blocking the NOD1 receptor causes symptoms of stress-induced anxiety, cognitive impairment and depression [87]. *Lactobacillus plantarum* Lac16 increased NOD1 expression level [35]. In the current study, Bactolac was not effective in increasing NOD1 expression levels in stressful situation.

Clinical and preclinical studies have shown that apoptosis which has an important role in neurodegeneration and death of glial cells, contributes to mental diseases and especially stress-induced depression. Additionally, apoptosis reduces the effectiveness of antidepressant drugs [22, 32, 130]. Chronic stress stimulates apoptosis in the cerebral cortex and increases the expression levels of caspase proteins [7]. Chronic stress causes neuronal apoptosis and increased Caspase- 3 expression level [39]. Microglia cells which can be stimulated by various cytokines, modulators, neurotransmitters, neurotoxins, extracellular matrix molecules and proteases, are very active and versatile. Stress, a common factor in the development of depression and depressive-like behaviors, causes to activate the M1 phenotype of microglia and synthesize pro-inflammatory cytokines (IL- 6, IL- 1β and TNF-α) and oxidative mediators (ROS and NO), and these mediators are abundant in depressed patients [1, 52, 88]. Microglial cells are densely located in the hippocampus, and stress causes hippocampal microglial activation and thus inflammation, and these conditions often occur in depression. Excessive activation of microglia plays a role in the pathophysiology of depression; triggers or exacerbates depression [14, 19, 53]. Glial activity increases under the influence of stress and is often an indicator of degeneration and disruption of chemical balance. Glial activation is determined by Ki- 67 activity [79]. CUMS increased neuronal apoptosis and Caspase- 3 expression level in rats [39]. *Lactobacillus plantarum* 200655 decreased Caspase- 3 activity [85]. Emotional or physical stress caused neuroinflammation via microglial activation in rats [20]. *Lactobacillus plantarum* and *Bifidobacterium lactis* probiotic mix reduced Ki- 67 activity [101]. *Lactobacillus plantarum* PS128 reduced glial activity [114], and *Lactiplantibacillus plantarum* CR12 reduced CUMS-induced hippocampal microglial inflammation damage [71]. In our study, Bactolac was effective in reducing neurodegeneration and glial activity, caused antidepressant effects in stressful conditions. Psychological stress and the overproduction of pro-inflammatory cytokines lead to an increase in intestinal permeability, and disruption of intestinal permeability leads to intestinal dysbiosis. Increased permeability and dysbiosis are evident in depression and depression-related neuro-immune conditions. Approximately 60% of patients with anxiety and depression have intestinal dysfunction [47, 80]. *Lactobacillus plantarum* P8 and *Lactobacillus plantarum* ZLP001 strengthened intestinal barrier [112, 113]. In this study, Bactolac was not very effective in reducing intestinal permeability under stressful condition.

## Conclusion

Expression levels of 5-HT1 A, DRD1, ADRA- 2 A, GABA-A α1 and MC4R differed in the stress group compared to the control group, however, the fact that these differences were not statistically significant does not indicate that these differences are clinically insignificant. Histopathological results and behavioral tests prove our opinion. Neuron damage and glial activity increased in the stress group due to chronic stress. It was effective in reducing depressive-like behaviors according to the results of behavioral tests. Bactolac treatment provided high neuronal survival rate increasing BDNF level, prevented the excessive release of pro-inflammatory cytokines by reducing the expression levels of NLRP3 and MC4R, therefore, prevented the excessive activation of the HPA axis and accordingly, reduced neurodegeneration and glial cell activation in depressed rats. Bactolac provided antidepressant effects and also reduced depressive behaviors, as demonstrated by the results of four different behavioral tests in stressed rats. These findings suggest that Bactolac supplementation may be effective in coping with stress, alleviate the impacts of chronic stress and help to protect mental health. Further studies are warranted to explore the clinical potential of Bactolac in managing stress-related psychiatric disorders.

## Data Availability

The data used to support the findings of this study are available from the corresponding author upon request.
